# They Cannot, They Will Not, or We Are Asking the Wrong Questions: Re-examining Age-Related Decline in Social Cognition

**DOI:** 10.3389/fpsyg.2022.894522

**Published:** 2022-05-11

**Authors:** Lucas J. Hamilton, Amy N. Gourley, Anne C. Krendl

**Affiliations:** Department of Psychological and Brain Sciences, Indiana University, Bloomington, IN, United States

**Keywords:** social cognition and aging, motivation, research design, ecological validity, representative samples

## Abstract

Social cognition is critical for successfully navigating social relationships. Current evidence suggests that older adults exhibit poorer performance in several core social-cognitive domains compared to younger adults. Neurocognitive decline is commonly discussed as one of the key arbiters of age-related decline in social-cognitive abilities. While evidence supports this notion, age effects are likely attributable to multiple factors. This paper aims to recontextualize past evidence by focusing issues of motivation, task design, and representative samples. In light of these issues, we identify directions for future research to aide our understanding of social-cognitive aging.

## Introduction

Social connectedness has been widely implicated in preserving older adults’ cognitive, physical, and mental wellbeing (Shankar et al., 2011; Boss et al., 2015; Kuiper et al., 2015). Social-cognitive function—the process by which people understand, store, and apply information about others (Fiske and Taylor, 1991)—is essential for maintaining social connectedness (see Krendl and Heatherton, 2009), and relates to social relationships in later life (Krendl et al., 2022). Several key social-cognitive domains are disrupted by aging including emotion recognition—decoding another person’s feelings through non-verbal cues (Ruffman et al., 2008; Gonçalves et al., 2018; Hayes et al., 2020), impression formation—forming and managing impressions of others (Cassidy et al., 2016, 2020; Krendl and Kensinger, 2016; Krendl, 2018), and theory of mind—inferring the mental states of others (Henry et al., 2013; Moran, 2013). This paper outlines the support for several mechanisms that may contribute to age-related social-cognitive decline. However, it is not meant as a systematic or meta-analytic article as others have published such work (e.g., Demichelis et al., 2020; Hayes et al., 2020).

**Figure 1:**
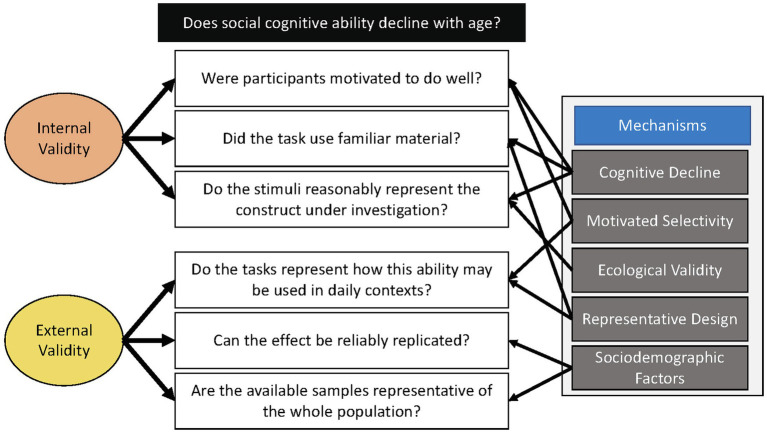
Questions for evaluating future research. Arrows represent if a mechanism relates to each question.

Although the preponderance of research on age-related social-cognitive decline has focused on declines in general cognitive ability, we argue that mechanisms are likely more nuanced. Over-emphasizing a single mechanism may limit potential interventions targets by overlooking key factors that contribute to deficits. To be forthcoming on our perspective, we provide Figure 1 as a consolidated visualization of the key arguments (formulated as questions), their ties to each proposed mechanism reviewed here, and their contributions to the design’s internal and external validity. Importantly, mechanisms are not mutually exclusive and may work in tandem (i.e., two mechanisms may answer the same question), but each provides its own unique strengths as an explanatory mechanism underlying social-cognitive processes across adulthood. Additionally, replicability concerns are not new and well-known across science (Ioannidis, 2005; Open Science Collaboration, 2012), and research must address generalizability by explicitly improving sample representativeness. We offer this paper as a re-examination of past research while focusing on motivation, study design, and sample representativeness and considering the threat to internal and/or external validity that each factor may pose.

## “They Cannot”: Links Between Cognitive Decline and Social-Cognitive Ability

Many studies have focused on the overlap between general cognitive and social-cognitive decline (see Moran, 2013; Gonçalves et al., 2018; Demichelis et al., 2020; Schlegel et al., 2020). Normative declines in fluid abilities (i.e., speed of processing, working memory, and executive function: see Salthouse, 2019) co-occur with declines in social-cognitive function (Sandoz et al., 2014; Moreau et al., 2016). For instance, inhibitory failures that arise from poorer executive functioning beget bias-prone responses during impression formation (e.g., Cassidy et al., 2016, 2020; Krendl and Kensinger, 2016; Krendl, 2018; Von Hippel, 2007). However, it should be noted that most social-cognitive aging studies are cross-sectional in nature, limiting any assessment of causality in the absence of longitudinal data.

Beyond evidence of concomitant age differences, there are many parallels between cognitive and social-cognitive aging research. Given that most social-cognitive tasks have time constraints or require coordinating multiple pieces of information (as is the case with cognitive aging tasks), it is unsurprising that performance is confounded between the two. Moreover, performance across social-cognitive domains (i.e., Theory of Mind and emotion recognition) appear to be scaffolded in a manner consistent with general cognitive models (see meta-analysis by Schurz et al., 2021). For instance, during mentalization for Theory of Mind, older adults may make more errors than younger adults due to poor mobilization of these underlying cognitive resources (e.g., Lecce et al., 2019). Thus, it could be that older adults perform worse on social-cognitive tasks due to decreased cognitive ability.

An important caveat to the above point is that social-cognitive performance can be maintained even in the presence of cognitive decline (see Krendl et al., 2014 or Strickland-Hughes et al., 2020 as examples). In fact, general cognition and social cognition engage distinct, albeit overlapping, patterns of neural activation (e.g., MacPherson et al., 2002; Schurz et al., 2014) even in neuropathological decline (e.g., Poletti et al., 2012; Belfort et al., 2020 for a review). Thus, at least some of the brain networks that underscore social cognition may be relatively resilient to age-related decline. Consistent with this assertion, neurological development in early childhood does not linearly predict the emergence of social-cognitive abilities (e.g., Tousignant et al., 2017; Meinhardt-Injac et al., 2020). With this as a reference point, it is perhaps unsurprising to suggest that normative neurological decline may not be associated with universal declines in all aspects of social cognition.

Moreover, there is evidence that suggests lab-based measures misrepresent the magnitude of age effects in social cognition. For instance, older adults are better at recognizing emotions during conversations with marital partners despite relatively poor performance on lab-based tasks (Sze et al., 2012). Rather than adopting a “they cannot” mindset that conflates social cognition with general cognition, we must identify how older adults’ performance may be driven by other factors that could occlude accurate estimation of their social-cognitive abilities.

## “They Will Not”: A Motivation-Based Account of Selective Engagement

Motivation in social-cognitive tasks may be shaped by myriad factors, including cognitive decline (e.g., Hess, 2006, 2014) or lifetime experiences (e.g., Carstensen et al., 1999). With respect to the former, social cognition is resource intensive, meaning it requires neurological and psychological resources (e.g., Fiske and Taylor, 1984). When resources are limited (e.g., due to cognitive decline), individuals may be less able or willing to expend them (e.g., Hess, 2006, 2014), which promotes selectivity in determining when and how to engage those resources. With respect to the latter, older adults may utilize experience-based social knowledge when deciding when and how to engage (e.g., Hess and Auman, 2001; Blanchard-Fields, 2007). Socioemotional selectivity theory (Carstensen et al., 1999) suggests that older adults are chronically motivated to prioritize familiar and close social partners. Older adults may expend more effort or utilize better strategies in such contexts, perhaps be getting better performance. Ultimately, varying age effects in past research may be due to older adults’ motivation rather than cognitive ability (as in cognitive aging: see Swirsky and Spaniol, 2019).

Evidence shows how explicitly motivating performance goals increases older adults’ performance. Indeed, age differences in emotion recognition and person perception disappear entirely when people are told that they will need to justify their judgments after completing the task (Hess et al., 2001, 2009a,b; Stanley and Isaacowitz, 2015), despite the fact that self-rated motivation does not influence emotion recognition (Ceccato et al., 2019). Thus, evidence of age-related decline in social cognition may be an artifact of older adults’ implicit resource preservation goals which leads to worse performance, especially when there are no consequences for being wrong. Yet, little work has tested if older adults will selectively modulate their level of effort based on explicit or implicit goals.

Certain factors (i.e., familiarity, wanting people to like you, and personal closeness) can increase older adults’ motivation. Own-age biases emerge for facial recognition and face–name associative memory tasks (Rhodes and Anastasi, 2012; Strickland-Hughes et al., 2020). Older adults better recognize emotions in their marital partners when compared to strangers (Sze et al., 2012). Theory of mind is greater among older adults who desire to be liked by others (Lecce et al., 2017) and when social closeness is experimental increased (Zhang et al., 2013, 2018). Conversely, older adults express more bias when perceiving members of out-groups, such as racial minorities, people who are homeless, and religious groups (Von Hippel et al., 2000; Cassidy et al., 2016, 2020; Krendl and Kensinger, 2016; Krendl, 2018). Altogether, these studies suggest that older adults can perform well but may choose not to.

We must identify factors that motivate older adults and if these factors are equally motivating for younger adults. If older adults are less motivated by traditional laboratory paradigms than younger adults, this calls into question the internal validity of many studies to date as well as the degree of generalizability and external validity. One way future research can test this motivational account is by examining the strategies that older adults use and their relative effectiveness. If older adults use more effortful strategies when motivated to perform well, then they may use less effortful strategies when unmotivated and perform worse as a result (see Hess et al., 2013). However, it will be important to interrogate whether effectively utilizing effortful strategies is predicated on cognitive abilities. For instance, theory of mind performance increases with strategy training (Cavallini et al., 2015, 2021; Lecce et al., 2015, 2019), but only when older adults have sufficient cognitive resources (e.g., executive function: see Lecce et al., 2019). Thus, future research will need to interrogate the limits of using motivation to improve to performance on social-cognitive tasks and explicitly evaluate factors that motivate older adults to perform well.

In evaluating what is (and is not) motivating for older adults, the field needs to re-evaluate our methodologies. However, this redirects our focus to the stimuli and tasks commonly deployed and exposes possible pitfalls. For instance, evidence using a face sort task (instead of prototypical forced-choice paradigms) shows that older and younger adults’ emotion recognition performance (i.e., assigning emotion identifiers to faces) is mostly comparable, but older adults are more nuanced in the emotion terms they use (Hoemann et al., 2021). In fact, consistent concerns have been raised regarding the stimuli and tasks that are used to assess social-cognitive abilities (e.g., Isaacowitz and Stanley, 2011; Freund and Isaacowitz, 2013; Kunzmann and Isaacowitz, 2017). Beyond motivation to perform, we must address the tasks themselves.

## Asking the Wrong Questions? the Limits of Current Tasks

Ecological validity in social cognition generally refers to the degree of the “realness” that the stimuli and tasks have. This usually hinges on two key dimensions: artificiality versus naturality and simplicity versus complexity (see Holleman et al., 2020 for discussion). However, ecological validity is often conflated with the issue of representative design—how effects translate from laboratory tasks to daily life contexts. Put simply, ecological validity refers to stimuli whereas representative design speaks to the context of the stimuli. Consequently, ecological validity is directly related to internal validity (i.e., testing the construct with appropriate items), whereas representative design addresses both internal and external validity as it represents the degree to which laboratory tasks mimic the phenomenon as it occurs in daily contexts. Although the former is more routinely discussed, the latter is a pressing issue that must be addressed for the field to move forward.

One of the main ecological validity concerns that has received the most attention is static versus dynamic stimuli. Differential age effects emerge depending on which is used (e.g., Hayes et al., 2020). For example, age-related differences in emotion recognition are less robust when using videos rather than pictures (e.g., Krendl and Ambady, 2010). However, the effects can be complex. That is, although dynamic stimuli (i.e., film clips) seem to increase performance (presumably due to their increased naturalness), they may simultaneously interfere with performance because they often increase the number of cues to be integrated (thereby increasing difficulty). This discordance is reflected in theory of mind research: age differences do not disappear when using dynamic stimuli (Henry et al., 2013), and, in some cases, may increase in magnitude (see Grainger et al., 2019, 2021). This illustrates the difficulty in defining the complexity and naturality of a task.

Instead, ecological validity should be evaluated in terms of how the stimuli (and how they are used in a task) plausibly represent the construct under investigation (see Holleman et al., 2020). Dynamic stimuli may be better at simulating a given phenomenon but offer less experimental control. To balance both, laboratory studies have leveraged virtual reality paradigms, which allow researchers to create a virtual social world while limiting what the participant can see and do (see Parsons, 2015). These paradigms appear to improve performance in younger adults with social-cognitive impairments (e.g., Kandalaft et al., 2013), and early evidence indicates that virtual reality interventions may promote assorted health-related benefits for older adults (see Dermody et al., 2020). Despite improving the accuracy of social-cognitive assessments (i.e., internal validity), concerns remain on how well findings replicate in other contexts (i.e., external validity). Poor representative design may provide incorrect conclusions about older adults’ social-cognitive ability in daily experiences.

Unfortunately, representative design has been underdiscussed in comparison to ecological validity despite evidence that lab-based tasks may underestimate social-cognitive ability (as described previously with regard to motivation as in Lecce et al., 2019 or Sze et al., 2012). It could be that daily social interactions in older adulthood are far more motivating and less demanding than any lab-based task. Consequently, the field must develop paradigms that evaluate social-cognitive processes in daily life. Ecological momentary assessments may help capture how people behave in more natural environments and have been used to capture the types of social interactions people have in daily life (e.g., Zhaoyang et al., 2018). However, such studies must be accessible to all and not overly complex to avoid inadvertently eliciting age effects (Cain et al., 2009). They also do not inherently solve the problem of evaluating social cognition during real interactions. Addressing representative design will require developing sensitive measures of daily social interactions, whether in the lab or daily life.

Ultimately, improving our measures is important as it will directly improve levels of internal and external validity in any given study. However, there is another major methodological concern that must be called out and specifically addressed in future research. The lack of representative samples is a looming blind spot in aging research at-large, and there may be important variability that is missed by overlooking sociodemographic factors and their relationship to social cognition.

## A Blind Spot in the Literature: Sample Representativeness

Sample representativeness in social-cognitive aging research must be addressed as differences are known to manifest when considering sociodemographic diversity (see Gutchess and Boduroglu, 2015). Evidence from an online study of over 40,000 people aged 10 to 70 revealed that theory of mind performance may be more sensitive to race, ethnicity, and education than it is to cognition (Dodell-Feder et al., 2020) as White, college-educated individuals perform the best. Thus, unaccounted variance across groups directly limits the external validity of past studies since a preponderance utilize White, middle-class, and college-educated samples.

Currently, 25% of people aged 65 or older in the United States identify as a member of a racial minority, which is expected to increase to 34% in the next two decades (Administration for Community Living, 2021). Extending the postulates of minority stress models (Forrester et al., 2019), older members of racial minority groups may have a lifetime of utilizing social resources to cope with multifaceted disadvantages (e.g., structural and interpersonal discrimination). Beginning as early as grade school (see Rowley et al., 2008), members of racial minorities may learn to be hypervigilant to out-group threats and mistrust unfamiliar social partners for self-preservation in the face of discrimination (see Brondolo et al., 2018). Members of racial minorities have a lifetime of maintaining these behaviors which may manifest patterns of social-cognitive aging that differ from members of racial majorities. Thus, one possibility is that older members of racial minorities may display age-related expertise in certain social situations. No direct evidence exists to evaluate the likelihood of this possibility, however, which only reiterates the need for future research.

Gender is another sociodemographic factor to consider. Some work has shown that women perform better than men on social-cognitive tasks, such as theory of mind (e.g., Wacker et al., 2017). These gender differences may be underscored by differences in strategy use (e.g., Adenzato et al., 2017, 2019). However, these differences may reflect how social cognition itself (i.e., understanding others’ thought, feelings, and behaviors) is a stereotypically feminine notion (see Martin and Slepian, 2021 for a discussion). Female gender roles of warmth and communality are reinforced from birth onward, presumably leading to social-cognitive differences that percolate and accumulate from adolescence into later life.

Future work should maintain an intersectional approach (see Collins and Bilge, 2020) to understand how membership in multiple groups may influence social cognition. As an example, Black women in the workforce often contend with several negative stereotypes due to their race and gender (see Collins, 2004). To face pervasive and systemic stereotypes throughout their lives, Black women may utilize an abundance of mindreading (i.e., Theory of Mind) to navigate their hypervisibility (Dickens et al., 2019) and avoid activating negative stereotypes associated with their identities (see Collins, 2004). Even with hypervigilant meta-cognitive awareness, they may still experience discrimination and microaggressions and utilize social-cognitive strengths (e.g., strong support networks) to cope (Holder et al., 2015). Thus, the lifelong utility of social-cognitive abilities may be best understood through an intersectional lens.

Social-cognitive aging research will benefit from exploring the role of sociodemographic factors. Special efforts need to be made to gather representative samples of older adults to interrogate the generalizability of social-cognitive aging effects and determine whether differential effects emerge within underrepresented populations (i.e., non-White, less educated, and low socioeconomic status). Thus, future research should examine sociodemographic factors, such as race and gender, to understand within-group variability for older populations. Ultimately, evidence garnered from this line of inquiry will be crucial for the advancement of the field.

## Concluding Remarks

Despite recent progress, many important questions remain. General cognitive decline appears to be concomitant with social-cognitive difficulties, but not all evidence supports this narrative. Other mechanisms, such as motivated selectivity, ecological validity, representative design, and sociodemographic factors should be further investigated. We believe that social-cognitive aging research has many paths forward including the development of new tasks and evaluating the day-to-day impact of social-cognitive function across adulthood. Although the current challenges require innovative solutions, the rich history of social-cognitive aging research suggests that researchers will meet these demands and continue to push the field into the future.

## Data Availability Statement

The original contributions presented in the study are included in the article/supplementary material, and further inquiries can be directed to the corresponding author.

## Author Contributions

LH lead the project by conducting literature searches and writing the manuscript. AG also collected relevant literature and wrote portions of initial drafts. AK provided conceptual guidance and critical feedback throughout the project. All authors contributed to the article and approved the submitted version.

## Funding

This work was supported by National Institute on Aging Grant R01 AG070931 (PI: AK).

## Conflict of Interest

The authors declare that the research was conducted in the absence of any commercial or financial relationships that could be construed as a potential conflict of interest.

## Publisher’s Note

All claims expressed in this article are solely those of the authors and do not necessarily represent those of their affiliated organizations, or those of the publisher, the editors and the reviewers. Any product that may be evaluated in this article, or claim that may be made by its manufacturer, is not guaranteed or endorsed by the publisher.

## References

[ref1] Adenzato M. Brambilla M. Manenti R. De Lucia L. Trojano L. Garofalo S.. (2017). Gender differences in cognitive theory of mind revealed by transcranial direct current stimulation on medial prefrontal cortex. Sci. Rep. 7, 1–9. doi: 10.1038/srep41219, PMID: 28117378 PMC5259730

[ref2] Adenzato M. Manenti R. Gobbi E. Enrici I. Rusich D. Cotelli M. (2019). Aging, sex and cognitive theory of mind: a transcranial direct current stimulation study. Sci. Rep. 9, 1–10. doi: 10.1038/s41598-019-54469-4, PMID: 31792263 PMC6889494

[ref3] Administration for Community Living (2021). 2020 Profile of Older Americans. Available at: https://acl.gov/sites/default/files/Profile%20of%20OA/2020ProfileOlderAmericans_RevisedFinal.pdf (Accessed March 11, 2022).

[ref4] Belfort T. Simões J. P. Santos R. L. Lacerda I. Dourado M. C. N. (2020). Social cognition: patterns of impairments in mild and moderate Alzheimer’s disease. Int. J. Geriatr. Psychiatry 35, 1385–1392. doi: 10.1002/gps.5379, PMID: 32662123

[ref5] Blanchard-Fields F. (2007). Everyday problem solving and emotion: An adult developmental perspective. Curr. Dir. Psychol. Sci. 16, 26–31. doi: 10.1111/j.1467-8721.2007.00469.x

[ref6] Boss L. Kang D. H. Branson S. (2015). Loneliness and cognitive function in the older adult: a systematic review. Int. Psychogeriatr. 27, 541–553. doi: 10.1017/S1041610214002749, PMID: 25554219

[ref7] Brondolo E. Blair I. V. Kaur A. (2018). “Biopsychosocial mechanisms linking discrimination to health: A focus on social cognition,” in The Oxford Handbook of Stigma, Discrimination, and Health. eds. Major B. Dovidio J. F. Link B. G. (England: Oxford University Press), 219–240.

[ref8] Cain A. E. Depp C. A. Jeste D. V. (2009). Ecological momentary assessment in aging research: a critical review. J. Psychiatr. Res. 43, 987–996. doi: 10.1016/j.jpsychires.2009.01.014, PMID: 19272611 PMC3638997

[ref9] Carstensen L. L. Isaacowitz D. M. Charles S. T. (1999). Taking time seriously: a theory of socioemotional selectivity. Am. Psychol. 54, 165–181. doi: 10.1037/0003-066X.54.3.165, PMID: 10199217

[ref10] Cassidy B. S. Hughes C. Lanie S. T. Krendl A. C. (2020). Effects of executive ability on bias and ingroup perceptions in aging. Psychol. Aging 35, 283–294. doi: 10.1037/pag0000420, PMID: 31647258 PMC7042085

[ref11] Cassidy B. S. Lee E. J. Krendl A. C. (2016). Age and executive ability impact the neural correlates of race perception. Soc. Cogn. Affect. Neurosci. 11, 1752–1761. doi: 10.1093/scan/nsw081, PMID: 27330185 PMC5091673

[ref12] Cavallini E. Bianco F. Bottiroli S. Rosi A. Vecchi T. Lecce S. (2015). Training for generalization in theory of mind: a study with older adults. Front. Psychol. 6:1123. doi: 10.3389/fpsyg.2015.01123, PMID: 26300818 PMC4523701

[ref13] Cavallini E. Ceccato I. Bertoglio S. Francescani A. Vigato F. Ianes A. B.. (2021). Can theory of mind of healthy older adults living in a nursing home be improved? A randomized controlled trial. Aging Clin. Exp. Res. 33, 3029–3037. doi: 10.1007/s40520-021-01811-4, PMID: 33682064 PMC8595145

[ref14] Ceccato I. Lecce S. Cavallini E. Vugt F. T. Ruffman T. (2019). Motivation and social-cognitive abilities in older adults: convergent evidence from self-report measures and cardiovascular reactivity. PLoS One 14:e0218785. doi: 10.1371/journal.pone.0218785, PMID: 31291276 PMC6619662

[ref15] Collins P. H. (2004). Black Sexual Politics: African Americans, Gender, and the New Racism. England: Routledge. 10.1080/13691058.2013.872316 24455983

[ref16] Collins P. H. Bilge S. (2020). Intersectionality. United States: John Wiley & Sons.

[ref17] Demichelis O. Coundouris S. Grainger S. Henry J. (2020). Empathy and theory of mind in Alzheimer’s disease: a meta-analysis. J. Int. Neuropsychol. Soc. 26, 963–977. doi: 10.1017/S1355617720000478, PMID: 32431261

[ref18] Dermody G. Whitehead L. Wilson G. Glass C. (2020). The role of virtual reality in improving health outcomes for community-dwelling older adults: systematic review. J. Med. Internet Res. 22:e17331. doi: 10.2196/17331, PMID: 32478662 PMC7296414

[ref19] Dickens D. D. Womack V. Y. Dimes T. (2019). Managing hypervisibility: an exploration of theory and research on identity shifting strategies in the workplace among black women. J. Vocat. Behav. 113, 153–163. doi: 10.1016/j.jvb.2018.10.008

[ref20] Dodell-Feder D. Ressler K. J. Germine L. T. (2020). Social cognition or social class and culture? On the interpretation of differences in social cognitive performance. Psychol. Med. 50, 133–145. doi: 10.1017/S003329171800404X 30616706

[ref21] Fiske S. T. Taylor S. E. (1984). Social Cognition. United States: Addison-Wesley.

[ref22] Fiske S. T. Taylor S. E. (1991). Social Cognition. New York: Mcgraw-Hill Book Company.

[ref23] Forrester S. N. Gallo J. J. Whitfield K. E. Thorpe R. J. Jr. (2019). A framework of minority stress: From physiological manifestations to cognitive outcomes. The Gerontologist 59, 1017–1023. doi: 10.1093/geront/gny104, PMID: 30169640 PMC6858824

[ref24] Freund A. M. Isaacowitz D. M. (2013). Beyond age comparisons: a plea for the use of a modified Brunswikian approach to experimental designs in the study of adult development and aging. Hum. Dev. 56, 351–371. doi: 10.1159/000357177

[ref25] Gonçalves A. R. Fernandes C. Pasion R. Ferreira-Santos F. Barbosa F. Marques-Teixeira J. (2018). Effects of age on the identification of emotions in facial expressions: a meta-analysis. PeerJ 6:e5278. doi: 10.7717/peerj.5278, PMID: 30065878 PMC6064197

[ref26] Grainger S. A. Rakunathan V. Adams A. G. Canty A. L. Henry J. D. (2021). An assessment of age differences in theory of mind using the virtual assessment of mentalizing ability. Aging Neuropsychol. Cognit. 28, 97–107. doi: 10.1080/13825585.2020.1713290, PMID: 31916892

[ref27] Grainger S. A. Steinvik H. R. Henry J. D. Phillips L. H. (2019). The role of social attention in older adults’ ability to interpret naturalistic social scenes. Q. J. Exp. Psychol. 72, 1328–1343. doi: 10.1177/1747021818791774, PMID: 30001675

[ref28] Gutchess A. H. Boduroglu A. (2015). “Cognition in adulthood across cultures,” in The Oxford Handbook of Human Development and Culture: An Interdisciplinary Perspective. ed. Jensen L. A. (England: Oxford University Press), 621–636.

[ref29] Hayes G. S. McLennan S. N. Henry J. D. Phillips L. H. Terrett G. Rendell P. G.. (2020). Task characteristics influence facial emotion recognition age-effects: a meta-analytic review. Psychol. Aging 35, 295–315. doi: 10.1037/pag0000441, PMID: 31999152

[ref30] Henry J. D. Phillips L. H. Ruffman T. Bailey P. E. (2013). A meta-analytic review of age differences in theory of mind. Psychol. Aging 28, 826–839. doi: 10.1037/a0030677, PMID: 23276217

[ref31] Hess T. M. (2006). Adaptive aspects of social cognitive functioning in adulthood: age–related goal and knowledge influences. Soc. Cogn. 24, 279–309. doi: 10.1521/soco.2006.24.3.279

[ref32] Hess T. M. (2014). Selective engagement of cognitive resources: motivational influences on older adults’ cognitive functioning. Perspect. Psychol. Sci. 9, 388–407. doi: 10.1177/1745691614527465, PMID: 26173272 PMC5911399

[ref33] Hess T. M. Auman C. (2001). Aging and social expertise: The impact of trait-diagnostic information on impressions of others. Psychol. Aging 16, 497–510. doi: 10.1037/0882-7974.16.3.497, PMID: 11554526

[ref34] Hess T. M. Germain C. M. Swaim E. L. Osowski N. L. (2009a). Aging and selective engagement: The moderating impact of motivation on older adults’ resource utilization. J. Gerontol. Ser. B Psychol. Sci. Soc. Sci. 64B, 447–456. doi: 10.1093/geronb/gbp020 PMC2697500 19357075

[ref35] Hess T. M. Leclerc C. M. Swaim E. Weatherbee S. R. (2009b). Aging and everyday judgments: the impact of motivational and processing resource factors. Psychol. Aging 24, 735–740. doi: 10.1037/a0016340, PMID: 19739930 PMC2742956

[ref37] Hess T. M. Queen T. L. Ennis G. E. (2013). Age and self-relevance effects on information search during decision making. J. Gerontol. B Psychol. Sci. Soc. Sci. 68, 703–711. doi: 10.1093/geronb/gbs108, PMID: 23197342 PMC3859358

[ref38] Hess T. M. Rosenberg D. C. Waters S. J. (2001). Motivation and representational processes in adulthood: the effects of social accountability and information relevance. Psychol. Aging 16, 629–642. doi: 10.1037/0882-7974.16.4.629, PMID: 11766917

[ref39] Hoemann K. Vicaria I. M. Gendron M. Stanley J. T. (2021). Introducing a face sort paradigm to evaluate age differences in emotion perception. J. Gerontol. B 76, 1272–1281. doi: 10.1093/geronb/gbaa038, PMID: 32211791 PMC8363038

[ref40] Holder A. M. Jackson M. Ponterotto J. (2015). Racial microaggression experiences and coping strategies of black women in corporate leadership. Qual. Psychol. 2, 164–180. doi: 10.1037/qup0000024

[ref41] Holleman G. A. Hooge I. T. Kemner C. Hessels R. S. (2020). The ‘real-world approach’ and its problems: A critique of the term ecological validity. Front. Psychol. 11:721. doi: 10.3389/fpsyg.2020.00721, PMID: 32425850 PMC7204431

[ref42] Ioannidis J. P. (2005). Why most published research findings are false. PLoS Med. 2:e124. doi: 10.1371/journal.pmed.0020124, PMID: 16060722 PMC1182327

[ref43] Isaacowitz D. M. Stanley J. T. (2011). Bringing an ecological perspective to the study of aging and recognition of emotional facial expressions: past, current, and future methods. J. Nonverbal Behav. 35, 261–278. doi: 10.1007/s10919-011-0113-6, PMID: 22125354 PMC3223963

[ref44] Kandalaft M. R. Didehbani N. Krawczyk D. C. Allen T. T. Chapman S. B. (2013). Virtual reality social cognition training for young adults with high-functioning autism. J. Autism Dev. Disord. 43, 34–44. doi: 10.1007/s10803-012-1544-6, PMID: 22570145 PMC3536992

[ref47] Krendl A. C. (2018). Reduced cognitive capacity impairs the malleability of older adults’ negative attitudes to stigmatized individuals. Exp. Aging Res. 44, 271–283. doi: 10.1080/0361073X.2018.1475152, PMID: 29781770

[ref48] Krendl A. C. Ambady N. (2010). Older adults’ decoding of emotions: role of dynamic versus static cues and age-related cognitive decline. Psychol. Aging 25, 788–793. doi: 10.1037/a0020607, PMID: 21186915

[ref50] Krendl A. C. Heatherton T. F. (2009). “Self versus others/self-regulation,” in Handbook of Neuroscience for the Behavioral Sciences. eds. Bernston G. G. Cacioppo J. T. (New Jersy: John Wiley & Sons, Inc.), 859–878.

[ref51] Krendl A. C. Kennedy D. P. Hugenberg K. Perry B. L. (2022). Social cognitive abilities predict unique aspects of older adults’ personal social networks. J. Gerontol. B 77, 18–28. doi: 10.1093/geronb/gbab048, PMID: 33733655 PMC8755914

[ref52] Krendl A. C. Kensinger E. A. (2016). Does older adults’ cognitive function disrupt the malleability of their attitudes toward outgroup members?: An fMRI investigation. PLoS One 11:e0152698. doi: 10.1371/journal.pone.0152698, PMID: 27074046 PMC4830528

[ref53] Krendl A. C. Rule N. O. Ambady N. (2014). Does aging impair first impression accuracy? Differentiating emotion recognition from complex social inferences. Psychol. Aging 29, 482–490. doi: 10.1037/a0037146, PMID: 25244469

[ref54] Kuiper J. S. Zuidersma M. Voshaar R. C. O. Zuidema S. U. van den Heuvel E. R. Stolk R. P.. (2015). Social relationships and risk of dementia: A systematic review and meta-analysis of longitudinal cohort studies. Ageing Res. Rev. 22, 39–57. doi: 10.1016/j.arr.2015.04.006, PMID: 25956016

[ref55] Kunzmann U. Isaacowitz D. (2017). Emotional aging: taking the immediate context seriously. Res. Hum. Dev. 14, 182–199. doi: 10.1080/15427609.2017.1340048

[ref56] Lecce S. Bottiroli S. Bianco F. Rosi A. Cavallini E. (2015). Training older adults on theory of mind (ToM): transfer on metamemory. Arch. Gerontol. Geriatr. 60, 217–226. doi: 10.1016/j.archger.2014.10.001, PMID: 25456890

[ref57] Lecce S. Ceccato I. Bianco F. Rosi A. Bottiroli S. Cavallini E. (2017). Theory of mind and social relationships in older adults: the role of social motivation. Aging Ment. Health 21, 253–258. doi: 10.1080/13607863.2015.1114586, PMID: 26581839

[ref58] Lecce S. Ceccato I. Cavallini E. (2019). Investigating ToM in aging with the MASC: from accuracy to error type. Aging Neuropsychol. Cognit. 26, 541–557. doi: 10.1080/13825585.2018.1500996, PMID: 30041573

[ref59] MacPherson S. E. Phillips L. H. Della Sala S. (2002). Age, executive function and social decision making: a dorsolateral prefrontal theory of cognitive aging. Psychol. Aging 17, 598–609. doi: 10.1037/0882-7974.17.4.598, PMID: 12507357

[ref60] Martin A. E. Slepian M. L. (2021). The primacy of gender: gendered cognition underlies the big two dimensions of social cognition. Perspect. Psychol. Sci. 16, 1143–1158. doi: 10.1177/1745691620904961, PMID: 32516068

[ref61] Meinhardt-Injac B. Daum M. M. Meinhardt G. (2020). Theory of mind development from adolescence to adulthood: testing the two-component model. Br. J. Dev. Psychol. 38, 289–303. doi: 10.1111/bjdp.12320, PMID: 31960462

[ref62] Moran J. M. (2013). Lifespan development: The effects of typical aging on theory of mind. Behav. Brain Res. 237, 32–40. doi: 10.1016/j.bbr.2012.09.020, PMID: 23000532

[ref64] Moreau N. Rauzy S. Viallet F. Champagne-Lavau M. (2016). Theory of mind in Alzheimer disease: evidence of authentic impairment during social interaction. Neuropsychology 30, 312–321. doi: 10.1037/neu0000220, PMID: 26146852

[ref65] Open Science Collaboration (2012). An open, large-scale, collaborative effort to estimate the reproducibility of psychological science. Perspect. Psychol. Sci. 7, 657–660. doi: 10.1177/1745691612462588, PMID: 26168127

[ref66] Parsons T. D. (2015). Virtual reality for enhanced ecological validity and experimental control in the clinical, affective and social neurosciences. Front. Hum. Neurosci. 9:660. doi: 10.3389/fnhum.2015.00660, PMID: 26696869 PMC4675850

[ref67] Poletti M. Enrici I. Adenzato M. (2012). Cognitive and affective theory of mind in neurodegenerative diseases: neuropsychological, neuroanatomical and neurochemical levels. Neurosci. Biobehav. Rev. 36, 2147–2164. doi: 10.1016/j.neubiorev.2012.07.004, PMID: 22819986

[ref68] Rhodes M. G. Anastasi J. S. (2012). The own-age bias in face recognition: a meta-analytic and theoretical review. Psychol. Bull. 138, 146–174. doi: 10.1037/a0025750, PMID: 22061689

[ref69] Rowley S. J. Burchinal M. R. Roberts J. E. Zeisel S. A. (2008). Racial identity, social context, and race-related social cognition in African Americans during middle childhood. Dev. Psychol. 44, 1537–1546. doi: 10.1037/a0013349, PMID: 18999320

[ref70] Ruffman T. Henry J. D. Livingstone V. Phillips L. H. (2008). A meta-analytic review of emotion recognition and aging: implications for neuropsychological models of aging. Neurosci. Biobehav. Rev. 32, 863–881. doi: 10.1016/j.neubiorev.2008.01.001, PMID: 18276008

[ref71] Salthouse T. A. (2019). Trajectories of normal cognitive aging. Psychol. Aging 34, 17–24. doi: 10.1037/pag0000288, PMID: 30211596 PMC6367038

[ref72] Sandoz M. Démonet J. F. Fossard M. (2014). Theory of mind and cognitive processes in aging and Alzheimer type dementia: a systematic review. Aging Ment. Health 18, 815–827. doi: 10.1080/13607863.2014.899974, PMID: 24697253

[ref73] Schlegel K. Palese T. Mast M. S. Rammsayer T. H. Hall J. A. Murphy N. A. (2020). A meta-analysis of the relationship between emotion recognition ability and intelligence. Cognit. Emot. 34, 329–351. doi: 10.1080/02699931.2019.1632801, PMID: 31221021

[ref74] Schurz M. Radua J. Aichhorn M. Richlan F. Perner J. (2014). Fractionating theory of mind: a meta-analysis of functional brain imaging studies. Neurosci. Biobehav. Rev. 42, 9–34. doi: 10.1016/j.neubiorev.2014.01.009, PMID: 24486722

[ref75] Schurz M. Radua J. Tholen M. G. Maliske L. Margulies D. S. Mars R. B.. (2021). Toward a hierarchical model of social cognition: A neuroimaging meta-analysis and integrative review of empathy and theory of mind. Psychol. Bull. 147, 293–327. doi: 10.1037/bul0000303, PMID: 33151703

[ref76] Shankar A. McMunn A. Banks J. Steptoe A. (2011). Loneliness, social isolation, and behavioral and biological health indicators in older adults. Health Psychol. 30, 377–385. doi: 10.1037/a0022826, PMID: 21534675

[ref78] Stanley J. T. Isaacowitz D. M. (2015). Caring more and knowing more reduces age-related differences in emotion perception. Psychol. Aging 30, 383–395. doi: 10.1037/pag0000028, PMID: 26030775 PMC4451607

[ref79] Strickland-Hughes C. M. Dillon K. E. West R. L. Ebner N. C. (2020). Own-age bias in face-name associations: evidence from memory and visual attention in younger and older adults. Cognition 200:104253. doi: 10.1016/j.cognition.2020.104253, PMID: 32192981

[ref80] Swirsky L. T. Spaniol J. (2019). Cognitive and motivational selectivity in healthy aging. Wiley Interdiscip. Rev. Cogn. Sci. 10:e1512. doi: 10.1002/wcs.1512, PMID: 31183981

[ref81] Sze J. A. Goodkind M. S. Gyurak A. Levenson R. W. (2012). Aging and emotion recognition: not just a losing matter. Psychol. Aging 27, 940–950. doi: 10.1037/a0029367, PMID: 22823183 PMC3746016

[ref82] Tousignant B. Eugène F. Jackson P. L. (2017). A developmental perspective on the neural bases of human empathy. Infant Behav. Dev. 48, 5–12. doi: 10.1016/j.infbeh.2015.11.006, PMID: 26995647

[ref83] Von Hippel W. (2007). Aging, executive functioning, and social control. Curr. Dir. Psychol. Sci. 16, 240–244. doi: 10.1111/j.1467-8721.2007.00512.x

[ref84] Von Hippel W. Silver L. A. Lynch M. E. (2000). Stereotyping against your will: the role of inhibitory ability in stereotyping and prejudice among the elderly. Personal. Soc. Psychol. Bull. 26, 523–532. doi: 10.1177/0146167200267001

[ref85] Wacker R. Bölte S. Dziobek I. (2017). Women know better what other women think and feel: gender effects on mindreading across the adult life span. Front. Psychol. 8:1324. doi: 10.3389/fpsyg.2017.01324, PMID: 28824503 PMC5539187

[ref87] Zhang X. Fung H. H. Stanley J. T. Isaacowitz D. M. Ho M. Y. (2013). Perspective taking in older age revisited: a motivational perspective. Dev. Psychol. 49, 1848–1858. doi: 10.1037/a0031211, PMID: 23276131

[ref88] Zhang X. Lecce S. Ceccato I. Cavallini E. Zhang L. Chen T. (2018). Plasticity in older adults’ theory of mind performance: the impact of motivation. Aging Ment. Health 22, 1592–1599. doi: 10.1080/13607863.2017.1376313, PMID: 28885057

[ref89] Zhaoyang R. Sliwinski M. J. Martire L. M. Smyth J. M. (2018). Age differences in adults’ daily social interactions: an ecological momentary assessment study. Psychol. Aging 33, 607–618. doi: 10.1037/pag0000242, PMID: 29708385 PMC6113687

